# The value of NSE to predict ICU mortality in patients with septic shock: A prospective observational study

**DOI:** 10.1097/MD.0000000000030941

**Published:** 2022-10-07

**Authors:** Li-Tao Zhang, Xin Xu, Hu Han, Shu-Min Cao, Ling-Ling Li, Jian Lv, Li-Ru Zhang, Jian-Guo Li

**Affiliations:** a Department of Emergency, Hebei General Hospital, Shijiazhuang Hebei, China; b Graduate School of Hebei Medical University, Shijiazhuang Hebei, China; c Department of Oncology, Hebei General Hospital, Shijiazhuang Hebei, China.

**Keywords:** ICU mortality, neuron-specific enolase, septic shock

## Abstract

To investigate the predictive value of neuron-specific enolase (NSE) on intensive care unit (ICU) mortality in patients with septic shock.

Seventy-five patients with septic shock hospitalized in the emergency intensive care unit (EICU) of Hebei General Hospital from March 2020 to September 2021 were included, and the patients’ baseline characteristics and laboratory findings were collected. NSE levels on the first and fourth days after admission were retrieved. NSE% [(NSEday1 – NSEday4)/NSEday1 × 100%] and δNSE (NSEday1 – NSEday4) were calculated. The outcome indicator was ICU mortality. The patients were divided into the survivors group (n = 57) and the nonsurvivors group (n = 18). Multivariate analysis was performed to assess the relationship between NSE and ICU mortality. The predictive value of NSE was evaluated using receiver operating characteristic (ROC) curve.

There were no significant differences in age, gender, systolic blood pressure (SBP), heart rate (HR), acute physiology and chronic health evaluation II score (APACHE II score), source of infection, and comorbidities between the 2 groups (all *P* > .05). Interleukin-6 (IL-6), NSE (day1), and NSE (day4) were significantly higher in patients in the nonsurvivors group (all *P* < .05), and there were no statistical differences in other laboratory tests between the 2 groups (all *P* > .05). APACHE II score, IL-6, lactate (Lac), total bilirubin (TBil), NSE (day1), and NSE (day4) showed a weak positive correlation with ICU mortality in patients with septic shock (all *P* < .05). Multivariate logistic regression analysis demonstrated that APACHE II score (odds ratio [OR] = 1.166, 95% confidence interval [95% confidence interval [CI]] 1.005–1.352, *P* = .042), IL-6 (OR = 1.001, 95% CI 1.000–1.001, *P* = .003) and NSE (day4) (OR = 1.099, 95% CI 1.027–1.176, *P* = .006) were independently associated with the ICU mortality of sepsis shock patients. The area under the curve (AUCs) of APACHE II score, IL-6, NSE (day1), and NSE (day4) for predicting prognosis were 0.650, 0.694, 0.758 and 0.770, respectively (all *P* < .05). NSE(day4) displayed good sensitivity and specificity (Sn = 61.11%, Sp = 91.23%) for predicting ICU mortality with a cutoff value of 25.94 ug/L.

High-level NSE (day4) is an independent predictor of ICU mortality in sepsis shock patients, which may become a good alternate option for evaluating sepsis severity. More extensive studies are needed in the future to demonstrate the prognosis value of NSE.

## 1. Introduction

Sepsis is a clinical syndrome in which the host develops a systemic inflammatory response to infection^[1]^ and is an increasing cause of admission to the emergency department (ED). It can quickly grow into septic shock and multiple organ dysfunction syndromes (MODS) if not treated on time, and its fatality rate can be as high as 28 to 56%.^[2]^ Pathophysiological derangements occurring during sepsis, such as endothelial dysfunction, increased nitric oxide and arachidonic acid derivative synthesis, and activation of inflammatory patterns, are responsible for the dysregulated host response and development of organ damage.^[3]^ Existing studies recognize that immune dysfunction caused by uncontrolled systemic inflammatory response is the primary pathophysiological mechanism of sepsis.^[4]^ Many cells are involved, including endothelial cells and leukocytes, and multiple proinflammatory and anti-inflammatory mediators (cytokines, oxygen-free radicals, coagulation factors, and so forth). Although the understanding of sepsis and septic shock has increased continuously and medical technology has improved rapidly in recent years, the death rate of patients remains high due to the combined effects of disorders in circulation and organ dysfunction. It is, therefore, necessary to pay close attention to assess the outcome of the disease. Death-risk stratification in septic patients enables early identification of patients at high risk of death and facilitates rational allocation of medical resources to improve results.

The presentation of sepsis is highly dependent on the organ systems affected, which might include the heart,^[5]^ lungs,^[6]^ central nervous system (CNS),^[7]^ and several others, as seen in sepsis-induced MODS. Sepsis-associated encephalopathy (SAE) is a poorly understood acute cerebral dysfunction that appears in the setting of sepsis and septic shock, affecting as many as 71% of patients.^[8]^ As an indicator of sepsis, diagnosis of SAE occurs primarily through the detection of abnormalities in electroencephalogram recordings and abnormal mental status, along with clinical history, physical examination, laboratory tests, and neuroimaging evaluation.^[9]^ Neuron-specific enolase (NSE) is a glycolytic enzyme mainly expressed in neurons and glial cells. It is also found in neuroendocrine cells,^[10]^ neuroendocrine tumors and red blood cells.^[11]^ NSE has been applied as a biomarker for the differential diagnosis of small cell lung cancer.^[12]^ It also has a specific predictive value for the prognosis of patients with severe traumatic brain injury.^[13]^ Previous studies have found that sepsis, septic shock and SAE, rather than traumatic brain injury, can also cause an increase in NSE.^[14,15]^ Nevertheless, the value of NSE in predicting intensive care unit (ICU) mortality in septic shock patients is not clear. Therefore, we collected clinical data to study the clinical significance of NSE in determining the prognosis in patients with septic shock.

## 2. Materials and Methods

### 2.1. Study protocol

This prospective, observational, single-center study was conducted in accordance with the principles of the Declaration of Helsinki. It was approved by the Hebei General Hospital Ethics Committee (NO.2020003) on January 24, 2020. Patients with septic shock were enrolled who were hospitalized in the emergency intensive care unit (EICU) of Hebei General Hospital from March 2020 to September 2021. The sepsis and septic shock diagnosis criteria were based on the Third International Consensus Definitions for Sepsis and Septic Shock (Sepsis-3).^[16]^ The inclusion criteria included the following: age ≥ 18 years old, diagnosed with septic shock when admitted to EICU, and the length of ICU stay for over 4 days. The exclusion criteria were as follows: patients with malignant tumor, primary immunodeficiency or immunosuppressant therapy; patients with decompensated cirrhosis, hereditary diseases, congenital metabolic diseases, or the end stage of other chronic diseases with organ dysfunction; patients with brain injury (head trauma, cerebral stroke, intracranial infection, epilepsy and so on); patients with cardiac arrest and return of spontaneous circulation; hemolysis and hematologic diseases. A total of 106 consecutive critically ill patients with new-onset septic shock were admitted to the EICU during the study period. After excluding 31 patients, according to the pre-specified exclusion criteria, we included 75 patients. All enrolled patients received standard treatment during their stay in the EICU.^[17]^

### 2.2. Data collection

Clinical data obtained from electronic medical records included the first diagnosis, demographic data, underlying diseases, and infection source on admission to the EICU. We also collected heart rate (HR), systolic blood pressure (SBP), white blood cell count, platelet count, lactate (Lac), serum creatinine (Scr), total bilirubin (TBIL), albumin (Alb), D-dimer, procalcitonin (PCT), C-reactive protein (CRP), serum amyloid A (SAA) and interleukin-6 (IL-6) levels within the first 12 hour of EICU admission. The blood samples of NSE were collected in the mornings of the first [NSE (day1)] and fourth days [NSE (day4)] after EICU admission. NSE levels in serum samples were evaluated by an electrochemiluminescence assay kit (ECLIA, Roche Diagnostics, USA). NSE% [(NSEday1 – NSEday4)/NSEday1 × 100%] and δNSE (NSEday1 – NSEday4) were calculated. Acute physiologic assessment and chronic health evaluation II score (APACHE II score) was used to assess the severity of illness. All eligible patients were categorized into 2 groups according to their ICU mortality.

### 2.3. Statistical analysis

The data were processed and analyzed by SPSS for Windows, version 26.0 (SPSS Inc., Chicago, IL). A 2-sided *P* value ≤.05 was considered statistically significant. Continuous data are expressed as mean ± standard deviation ($\bar{X}\pm S$), and independent samples t test was used to compare means between groups. Non-normally distributed continuous variables were expressed as median (first/third quartile) (M[Q_L_, Q_U_]), and the Mann–Whitney U test was used for the comparison of means between groups. Spearman correlation test was used to assess correlation. The counting data were expressed by frequency and rate, and the comparison between groups was performed by Chi-square test or Fisher exact test. Those statistically significant in the univariate analysis were entered into a multivariate logistic regression model to predict ICU mortality.

MedCalc 12.7.0 software was used to calculate the receiver operating characteristic (ROC) curve and the area under the curve (area under the curve [AUC]). Z test was used to compare the 2 AUCs. The Youden index was calculated, and the value at the maximum Youden index was used as the cutoff value. The sensitivity, specificity, positive likelihood ratio, and negative likelihood ratio were determined according to the cutoff value.

We used the highest detection threshold for statistical analysis if results were above the detection limits.

## 3. Results

### 3.1. Comparison of baseline characteristics and laboratory findings of the patients between 2 groups

A total of 75 patients was screened for this study. There were no statistically significant differences in age, gender, SBP, HR, source of infection, and comorbidities between the 2 groups (all *P* > .05). APACHE II score was higher in patients in the nonsurvivors group than in the survivors group, but there was no statistical difference (*P* = .056). IL-6, NSE (day1) and NSE (day4) were significantly higher in patients in the nonsurvivors group than in the survivors group (all *P* < .05), and there were no statistical differences in other laboratory tests between the 2 groups (all *P* > .05) (Table 1).

**Table 1:** Baseline characteristics and laboratory findings of the patients. [$\bar{X}\pm S$, M(Q_L_, Q_U_), n(%)].

	Survivors(n = 57)	Nonsurvivors (n = 18)	*P*
**Baseline characteristics**			
Age (yrs)	71.46 ± 14.66	75.72 ± 13.38	.276
Sex (male, %)	41(71.9)	13(72.2)	.981
APACHE II score	18(15,23)	20.5(16.5,25.5)	.056
**Vital signs**			
SBP (mm Hg)	119.35 ± 24.69	107.22 ± 18.39	.059
HR (per min)	102.68 ± 17.25	105.06 ± 21.31	.633
**Source of infection**			
Pneumonia (n,%)	53(93.0)	16(88.9)	.626
UTI (n,%)	4(7.0)	0(0.0)	.567
Biliary tract (n,%)	0(0.0)	2(11.1)	.055
**Comorbidities**			
Hypertension (n,%)	33(57.9)	13(72.2)	.277
Diabetes (n,%)	18(31.6)	6(33.3)	.889
Renal disease (n,%)	1(1.8)	1(5.6)	.425
Coronary artery disease (n,%)	12(21.1)	7(38.9)	.212
COPD (n,%)	2(3.5)	0(0.0)	1.000
Cerebrovascular disease (n,%)	37(64.9)	11(61.1)	.770
Hepatic disease (n,%)	3(5.3)	0(0.0)	1.000
**Laboratory tests**			
PCT (ng/mL)	2.69(0.60,16.00)	7.20(0.48,26.29)	.660
CRP (mg/L)	166.46 ± 123.53	187.86 ± 92.85	.501
IL-6 (pg/mL)	143.20(53.76,543.05)	661.90(89.01,4337.00)	.014
SAA (mg/L)	410.45(254.78,976.91)	378.34(216.61,471.33)	.365
Neutrophil (×10^9^/L)	9.69(5.93,11.86)	14.05(7.15,20.37)	.054
Lymphocyte (×10^9^/L)	0.76(0.41,1.22)	0.47(0.28,1.16)	.283
Platelet (×10^9^/L)	180.00(129.00,264.00)	148.50(75.25,217.50)	.142
D-dimer (mg/L)	3.62(1.64,5.98)	2.80(1.40,7.90)	.823
Lac (mmol/L)	2.95(1.60,3.87)	4.05(1.98,7.30)	.111
Alb (g/L)	30.60(26.70,33.00)	31.10(26.50,32.30)	.862
TBil (μmol/L)	16.20(12.35,24.25)	20.00(10.95,34.25)	.327
Scr (μmol/L)	83.20(58.60,131.05)	116.20(60.00,204.48)	.297
NSE (day1)(ug/L)	17.98(13.36,23.51)	30.33(19.61,46.50)	.001
NSE (day4) (ug/L)	14.66(10.50,20.72)	28.58(14.83,40.62)	.001
NSE %	18.14(–6.68,40.32)	12.31(–5.83,38.27)	.673
δNSE(ug/L)	3.22(–0.77,8.53)	3.17(–1.81,16.01)	.664

Alb = albumin, APACHE II score = acute physiology and chronic health evaluation II score, COPD = chronic obstructive pulmonary disease, CRP = C-reactive protein, HR = heart rate, IL-6 = interleukin-6, Lac = lactate, NSE = neuron-specific enolase, PCT = procalcitonin, QL, QU = first/third quartile, SAA = serum amyloid A, SBP = systolic blood pressure, Scr = serum creatinine, TBIL = total bilirubin, UTI = urinary tract infection.

### 3.2. Spearman correlation analysis of factors associated with ICU mortality in patients with septic shock

APACHE II score, IL-6, Lac, TBil, NSE (day1), and NSE (day4) showed a weak positive correlation with ICU mortality in patients with septic shock (all *P* < .05) (Table 2).

**Table 2:** Spearman analysis of factors associated with ICU mortality in patients with septic shock.

	*r*	*P*
APACHE II score	0.246	.033
PCT (ng/mL)	0.029	.802
CRP (mg/L)	0.079	.501
IL-6 (pg/mL)	0.421	<.001
SAA (mg/L)	–0.164	.161
Neutrophil (×10^9^/L)	0.223	.055
Lymphocyte (×10^9^/L)	–0.114	.329
Platelet (×10^9^/L)	–0.182	.118
D-dimer (mg/L)	–0.001	.994
Lac (mmol/L)	0.238	.040
Alb (g/L)	–0.027	.815
TBil (μmol/L)	0.255	.027
Scr (μmol/L)	0.070	.549
NSE (day1) (ug/L)	0.421	<.001
NSE (day4) (ug/L)	0.480	<.001
NSE %	–0.071	.546
δNSE (ug/L)	0.020	.862

Alb = albumin, APACHE II score = acute physiology and chronic health evaluation II score, CRP = C-reactive protein, ICU = intensive care unit, IL-6 = interleukin-6, Lac = lactate, NSE = neuron-specific enolase, PCT = procalcitonin, SAA = serum amyloid A, Scr = serum creatinine, TBIL = total bilirubin.

### 3.3. Results of multivariate logistic regression analysis between the survivors group and the nonsurvivors group

Multivariate analysis was performed for the following variables: APACHE II score, IL-6, NSE (day1), and NSE (day4) (Table 3). These results demonstrated that APACHE II score (odds ratio [OR] = 1.166, 95% confidence interval [95% confidence interval [CI]] 1.005–1.352, *P* = .042), IL-6 (OR = 1.001, 95% CI 1.000–1.001, *P* = .003) and NSE (day4) (OR = 1.099, 95% CI 1.027–1.176, *P* = .006) were independently associated with the ICU mortality of patients with septic shock.

**Table 3:** Results of multivariate analysis between the survivors group and the nonsurvivors group.

	B	SE	Wald	*P* value	OR	95% CI
NSE (day1) (ug/L)	0.033	0.024	1.886	.170	1.034	0.986–1.084
NSE (day4) (ug/L)	0.094	0.034	7.480	.006	1.099	1.027–1.176
IL-6(pg/mL)	0.001	0.000	9.085	.003	1.001	1.000–1.001
APACHE II score	0.153	0.076	4.119	.042	1.166	1.005–1.352

APACHE II score = acute physiology and chronic health evaluation II score, CI = confidence interval, IL-6 = interleukin-6, NSE = neuron-specific enolase, OR = odds ratio.

### 3.4. ROC curve analysis of APACHE II score, IL-6, and NSE (Day4) for discriminating survivors from nonsurvivors patients

**Figure 1.**
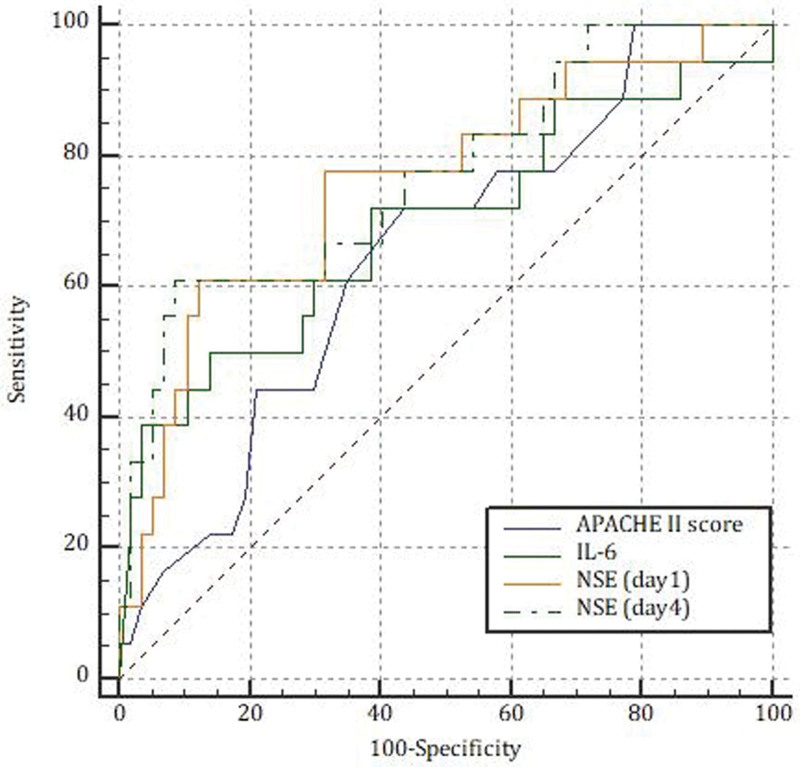
ROC curve analysis of APACHE II score, IL-6, NSE (day1) and NSE (day4) for predicting prognosis in patients with septic shock.

The AUCs of APACHE II score, IL-6, NSE (day1), and NSE (day4) for predicting prognosis in patients with septic shock were 0.650 (95%CI 0.531–0.757), 0.694 (95%CI 0.577–0.795), 0.758 (95%CI 0.646–0.850) and 0.770 (95%CI 0.658–0.859), respectively (all *P* < .05). Pairwise comparisons did not show significant differences among the groups (all *P* > .05) (Fig. 1).

APACHE II score = acute physiology and chronic health evaluation II score, IL-6 = interleukin-6, NSE = neuron-specific enolase, ROC = receiver operating characteristic.

### 3.5. Optimal cutoff values and sensitivity and specificity of APACHE II score, IL-6, and NSE (Day4) for predicting ICU mortality in patients with septic shock (Table 4 )

## 4. Discussion

The main goal of this work was to investigate the predictive value of NSE in determining ICU mortality in patients with septic shock. Our main findings are IL-6 and NSE levels (day1 and day4) were significantly higher in patients in the nonsurvivors group than in the survivors group. However, there were no statistical differences between the 2 groups in the change value and ratio of the fourth and first days of NSE (δNSE and NSE%). APACHE II score, IL-6, Lac, TBil, NSE (day1 and day4) showed a weak positive correlation with ICU mortality in patients with septic shock. APACHE II score, IL-6, and NSE (day4) levels were identified as a significant and independent prognostic factor for ICU mortality after adjusting for potential contributory factors. The AUCs of APACHE II score, IL-6, and NSE (day4) for predicting prognosis were 0.650, 0.694, and 0.770, respectively. Our data demonstrated that NSE (day4) might become a good alternate option for death-risk stratification in septic shock patients.

**Table 4:** Optimal cutoff values and sensitivity and specificity of NSE, IL-6, and APACHE II score for predicting prognosis in patients with septic shock.

	Cutoff value	Sensitivity	Specificity	Positive likelihood ratio	Negative likelihood ratio
NSE (day1) (ug/L)	27.46	61.11 (95% CI: 35.7–82.7)	87.72 (95% CI: 76.3–94.9)	4.98	0.44
NSE (day4) (ug/L)	25.94	61.11 (95% CI: 35.7–82.7)	91.23 (95% CI: 80.7–97.1)	6.97	0.43
IL-6 (pg/mL)	869.7	50.00 (95% CI: 26.0–74.0)	85.96 (95% CI: 74.2–93.7)	3.56	0.58
APACHE II score	18	72.22 (95% CI: 46.5–90.3)	56.14 (95% CI: 42.4–69.3)	1.65	0.49

APACHE II score = acute physiology and chronic health evaluation II score, CI = confidence interval, IL-6 = interleukin-6, NSE = neuron-specific enolase.

In sepsis, the combination of systemic inflammatory factors is paramount for developing MODS. Brain damage in the form of SAE, or sepsis-associated delirium or sepsis-associated brain dysfunction, is one of the most frequent and early components of MODS in sepsis.^[18]^ Cerebrovascular impairment and neuroinflammation are the 2 main triggering mechanisms of SAE.^[15]^ Many other mechanisms are also likely to participate in the pathogenesis of SAE. Neuronal necrosis and apoptosis are thought to directly induce neuronal loss in the brain following lipopolysaccharide (LPS)-induced SAE.^[19]^ Moreover, mitochondrial dysfunction and increased reactive oxygen/nitrogen species can promote neuronal death.^[20]^ In SAE, astrocytes are present in astrogliopathic states, which can gain abnormal functions that facilitate the unfavorable course of neuroinflammation and brain dysfunction.^[21]^ In addition, Microglia can be activated by inflammatory mediators, adjacent cells and neurotransmitters in the acute phase of sepsis and then induce neuronal dysfunction in the brain.^[22]^ SAE has a wide range of potentially reversible cognitive manifestations, including reduced attention, disrupted sleep-wakefulness balance, impaired memory, speech, orientation, focal neurological deficits, seizure activity, and perception disorders (delusion-hallucinatory complex) terminating with a decreased consciousness and coma.^[23]^ The emergence of SAE in septic patients is a marker of the severity of the septic state, which increases the risk of death by 10%, necessitating distinct therapeutic approaches.^[16]^

NSE is a cytoplasmatic glycolytic pathway enzyme located within neurons and neuroectodermal cells. Neuronal damage and interrupted integrity of the blood-brain barrier, such as in SAE, can result in NSE release into cerebral spinal fluid and blood. NSE is a brain-derived protein extensively studied as peripheral biochemical markers for brain injury, especially neuron damage. Several studies showed a serum increase of NSE in 53% in patients with severe sepsis and septic shock.^[14]^ However, screening for NSE in SAE diagnosis is inconsistent with study results. In a prospective and observational study of 112 enrolled patients, NSE levels of 24.145 ng/mL were diagnostic for SAE with 82.8% specificity and 54.2% sensitivity, and NSE levels of 24.865 ng/mL were predictive of hospital mortality with 79.1% specificity and 46.7% sensitivity. AUC was 0.590 in septic patients.^[15]^ A recent study reported the diagnostic values for SAE of NSE and IL-6 on the third day were 14.36 μg/L and 91.305 mg/L with sensitivity 61.1%, 72.2% and specificity 73.9%, 69.6%, respectively. The diagnostic AUCs of NSE, IL-6, and NSE + IL-6 were 0.675, 0.709, and 0.774.^[24]^ However, the authors did not assess the predictive value for patient mortality. Recent studies indicate a higher specificity and sensitivity for increased detection of neurofilaments, especially the light chain of neurofilaments, in the course of SAE.^[25]^ The promising results of neurofilaments serum concentrations in sepsis and their predictive value for SAE need to be evaluated prospectively.

Our study found that increased levels of NSE predicted a poor outcome for septic shock patients, which could be explained by the following perspectives. Brain dysfunction is one of the most frequent organ dysfunction in septic shock patients. CNS damage and interrupted blood-brain barrier integrity can result in NSE release into the blood. Therefore, elevated serum levels of NSE may predict prognosis in patients with septic shock as a peripheral biochemical marker for brain injury. In our study, NSE on the first and fourth days can predict ICU mortality. However, the NSE level detected on the fourth day was an independent prognostic predictor and outperformed many conventional biomarkers and the NSE level detected on the first day. One of the highlights of the present study was evaluating the prognostic significance of the dynamic changes of NSE during the disease course. The levels of NSE decreased (δNSE) from the first day to the fourth day in both the nonsurvivors group and the survivors group (3.17 ug/L vs 3.22 ug/L), but it had no value in predicting ICU mortality. The change ratio in NSE (NSE%) was also used to evaluate prognosis, and there were no statistical differences between the two groups (12.31% vs 18.14%). Therefore, the absolute value of NSE had more clinical application value.

Consistent with previous findings, conventional biomarkers such as APACHE II score and IL-6 also had significant prognostic value in our study. APACHE II score is the most widely used and authoritative critical disease evaluation system. Disease assessment and prognosis prediction are often entirely accurate for common critical diseases. A retrospective study reported that the APACHE II score had excellent discriminative powers for predicting hospital mortality in septic patients (AUC = 0.80 95%CI 0.78–0.82).^[26]^ Huang et al reported AUC of APACHE II score for predicting 30-day survival in patients with sepsis was 0.680.^[27]^ Our findings were consistent with their results. However, previous studies have confirmed that the APACHE II score has certain defects for some diseases with strong specialty characteristics or special populations, conditions with special organ damage or abnormal physiological indicators.^[28]^ In addition, the APACHE II score requires many values and cannot be obtained quickly. IL-6 is essential in cell development, initiation of innate immunity and cell functions in adaptive immunity.^[29]^ IL-6 plays a crucial part in the systemic inflammatory response. Elevated IL-6 levels in plasma have been identified in septic patients and correlate with increased mortality.^[30]^ IL-6 was an independent prognostic predictor in our study, but the predictive ability was poor (AUC = 0.694).

PCT is a valuable biomarker of bacterial infection, and its use is associated with a reduced duration of antibiotic therapy in different clinical settings.^[31]^ In addition, PCT should be used to identify patients with poor prognoses. In our study, there were no statistical differences in PCT between the two groups (*P* > .05), which indicated that the absolute value of the initial PCT level had limited prognostic significance. PCT serum level trend must be analyzed over time. A blinded prospective multicenter observational clinical trial reported that the 28-day all-cause mortality was 2-fold higher when PCT did not show a decrease of more than 80% from baseline to day 4 (20% vs 10% *P* = .001).^[32]^ Lac is an indicator of tissue hypoperfusion and cell hypoxia and a key marker of mitochondrial dysfunction. The Lac level in patients with sepsis was higher in the death group than in the survival group, suggesting that Lac levels reflect the poor prognosis.^[33]^ However, in our study, there were no statistical differences between the two groups (*P* > .05). Clinically, Lac levels are usually monitored dynamically to assess the perfusion and the patient’s response to treatment and prognosis.

This study has some limitations. First, as a prospective single-center study, the sample size was small, but it is the most extensive study evaluating NSE in patients with septic shock at a two-time point. The clinical value of NSE needs to be further proven by multicenter randomized clinical trials with a larger sample size to reduce proportional error. Second, no healthy control and sepsis groups were included in this study, and there were no comparisons with septic shock patients.

## 5. Conclusions

In conclusion, APACHE II score, IL-6, and NSE were associated with the ICU mortality of patients with septic shock. NSE levels in septic shock patients might be easy-to-use disease markers in the EICU. Furthermore, NSE (day4) concentrations provide a promising prognostic biomarker related to ICU mortality.

## Acknowledgments

We would like to thank Li-tao Zhang, Xin Xu, Hu Han, Shu-min Cao, Ling-ling Li, Jian Lv, Li-ru Zhang, Jian-guo Li for their assistance and valuable discussion.

## Author contributions

**Data curation:** Shu-Min Cao, Ling-Ling Li, Jian Lv, Li-Ru Zhang.

**Investigation:** Xin Xu.

**Methodology:** Hu Han.

**Project administration:** Li-Tao Zhang.

**Writing – review & editing:** Jian-Guo Li.
